# Clinical Experience of Acupuncture Treatment for Non-Alcoholic Fatty Liver Disease

**DOI:** 10.1155/2022/2447529

**Published:** 2022-03-11

**Authors:** Kun-Da Hong, Tian Wan, Si-Yu Lu

**Affiliations:** Department of Rehabilitation Medicine, The Second Affiliated Hospital of Fujian Medical University, Quanzhou, Fujian, China

## Abstract

Based on the authors' clinical experience, the acupuncture treatment of non-alcoholic fatty liver disease mainly includes the following three aspects. (1) The etiology and pathogenesis of non-alcoholic fatty liver disease are based on “deficiency in origin and excess in superficiality.” The deficiency in origin means deficiency of the spleen and stomach, and the excess in superficiality is caused by hepatobiliary disorders. (2) The application of the theory of strengthening the spleen and mobilizing transportation should be considered for the treatment of non-alcoholic fatty liver disease by acupuncture and moxibustion. Therefore, the use of “treatment from the spleen” often has miraculous effects. (3) Skillful use of acupuncture, shallow acupuncture, acupoint thread embedding, and other traditional Chinese medicine therapies are used to regulate the liver and spleen. In addition, warm acupuncture is reused to warm the Yang and strengthen the body.

## 1. Introduction

Non-alcoholic fatty liver disease (NAFLD), also known as metabolic dysfunction-associated fatty liver disease (MAFLD), refers to the exclusion of alcohol and other clear liver damage factors. Clinicopathological syndromes in which diffuse hepatocyte bullous fat becomes the main feature include simple fatty liver disease and its evolution of steatohepatitis, fatty liver fibrosis, and cirrhosis [1]. Studies have shown that the prevalence of NAFLD is as high as 25.24% [2]. Relevant studies in recent years have shown that traditional acupuncture therapy has a positive role in the treatment of NAFLD, and its adverse reactions are mild, simple, and effectively treated [3]. The authors have accumulated experience in clinical acupuncture and moxibustion treatment of NAFLD, which is summarized as follows.

## 2. Etiology and Pathogenesis

**Figure 1:**
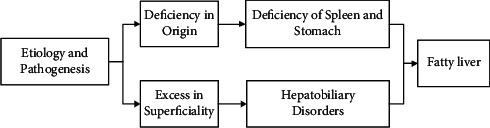
Etiology and pathogenesis of NAFLD.

The etiology and pathogenesis of non-alcoholic fatty liver disease are based on the “deficiency in origin and excess in superficiality”: the deficiency in origin refers to deficiency of the spleen and stomach, and the excess in superficiality is caused by stagnation of the liver and Qi (Figure 1).

### 2.1. Deficiency of the Spleen and Stomach Is the Root

Spleen and stomach are the acquired foundation. The spleen and stomach are weak in transporting water and grains, and the circulation of Qi and blood is not smooth, which leads to the deposition of phlegm and dampness in the liver and blood stasis in the liver. In disorders of the spleen and stomach, the turbidity is cleared without distinction, condensing the “cream” and forming fatty liver; hyperlipidemia occurs when turbid Qi enters the blood. Modern research has also shown that gastrointestinal function is closely related to NAFLD [4]. Research has found that an imbalance of the gastrointestinal flora is highly correlated with the progression of NAFLD. The imbalance of intestinal flora may affect the expression of an endogenous biologically active substance (adiponectin) secreted by adipocytes, leading to the occurrence of NAFLD [5].

### 2.2. Irregular Liver and Gallbladder Are the Targets

The liver is responsible for dredging and drainage. With the onset of disease, the liver is unbalanced, the Qi does not run smoothly, the Qi is blocked by phlegm, and bile excretion is not smooth. This worsens fatty liver. Modern medical research has also confirmed that disorders of lipid metabolism and hepatocyte steatosis are related to NAFLD [6, 7]. Human cells produce reactive oxygen species (ROS) during the metabolic process. There are more ROS in the livers of patients with fatty liver disease. Lipid peroxidation products and ROS increase and compromise mitochondrial membrane permeability, which eventually leads to liver cell apoptosis [8]. Excess lipid peroxidation products can directly lead to necrosis or apoptosis of liver cells. The process of peroxidation-necrosis directly promotes the development of liver fibrosis. On the other hand, the normal metabolism of bile acids is an important factor in ensuring normal glucose and lipid metabolism and the inhibition of liver fat accumulation. Disorders of bile acid metabolism affect liver glucose and lipid metabolism by changing the signalling pathway of TGR5 (a G-protein-coupled bile acid receptor). Therefore, bile acid metabolism is closely related to the formation and development of NAFLD [9].

## 3. Considering the Spleen for Treatment and Treating Disease from the Root

The pathogenesis of non-alcoholic fatty liver disease is led by spleen deficiency. Although the disease is located in the liver, it is inseparable from the spleen. Physiologically, the movement and transformation of the spleen depend on the maintenance of the Qi of the liver, and the dispersion of the liver and blood storage needs the spleen and stomach to metabolize and produce essence for nourishment. This reflects the roles and relationship of the liver and spleen in the function of dispersion and transportation and in blood production and blood storage. Regarding the meridian and collaterals, the “Lingshu·Meridian” points out that the spleen meridian “traces the tibia, then detours to the front of the Jueyin meridian,” and the liver meridian “is eight inches above the ankle and then detours to the back of the Taiyin meridian.” The Foot Taiyin Spleen Meridian and Foot Jueyin Liver Meridian cross and circulate on the inner side of the calf. The two meridians influence each other, which also fully reflects the close relationship between liver and spleen functions. In terms of pathology, Dongyuan Li mentioned in “Spleen and Stomach Theory” that “[with] internal injury to the spleen and stomach, all diseases arise.” If the spleen and stomach show insufficiency and dysfunction of transportation and chemistry, water dampness and turbid phlegm are inevitable; it can also affect liver failure and release, leading to the syndrome of “Tuyong Muyu.” Reflecting the pathological importance of the spleen, the signs and symptoms of NAFLD are mostly the manifestations of splenic failure. In the treatment of NAFLD, spleen strengthening should be the entry point [10, 11]. The spleen and stomach are the foundation of the acquired nature and the source of Qi and blood metaplasia, which can directly affect patient recovery and the outcome of the disease. The above shows that spleen deficiency is closely related to the occurrence and development of NAFLD, and the treatment should be based on the spleen.

## 4. Skillful Utilization of Acupuncture and Unique Acupoint Selection

**Figure 2:**
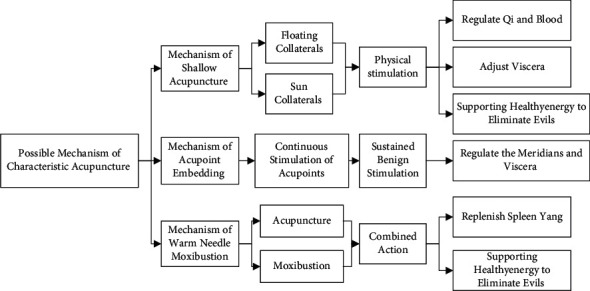
Possible mechanism of characteristic acupuncture.

The authors believe that acupuncture and moxibustion are effective in treating NAFLD, and they chose unique therapies—shallow acupuncture, acupoint embedding, and warm needle moxibustion. At the same time, the unique selection of acupoints can be effective (Figure 2).

### 4.1. Making Good Use of Shallow Needle Therapy

“Binghuang Wu's shallow acupuncture” has been included in the protection of intangible heritage. Shallow acupuncture can treat a variety of diseases, and its method of flattening, replenishing, and reducing is unique in the treatment of NAFLD. The principle of shallow acupuncture is to stimulate the meridian blood circulation through the physical stimulation of the meridian points by the needle tip, adjust the functional balance of the meridians and viscera, and play the role of strengthening the body, eliminating pathology and curing diseases [12, 13]. This is basically the same as the principle of acupuncture treatment. The difference is that shallow acupuncture produces a benign stimulus to the more sensitive floating collaterals and sun collaterals of the twelve skin regions with a gentle continuous tremor. Therefore, although the patient's local acupuncture sensation is not very obvious, the result depends on the amount of effective stimulation. In other words, it is no worse than the stimulation intensity produced by acupuncture and is mainly achieved by the duration and frequency of continuous effective stimulation. The weak tremor of shallow needles can produce resonance, which not only spreads to the surrounding area through the vibration wave centered on the meridian but also can follow the meridian circulation route in both directions to achieve the purpose of effective stimulation. *Treatment Method*. (1) Acupoint selection: Danzhong point. Point selection basis: Danzhong acupoint is the mu acupoint of the pericardium (the place where the Qi of the pericardium meridian gathers), the Qihui acupoint (the place where the Qi gathers), and the rendezvous point of the Ren channel, foot taiyin, foot shaoyin, hand taiyang, and hand shaoyang. The Danzhong point can regulate qi, activate blood and dredge the collaterals, and regulate the function of the Qi of the whole body. (2) Operation: the thumb is used to press the needle tail, the index finger and the middle finger to hold the needle handle, and the needle tip to press on the meridian skin. Use your middle finger nail to make continuous up and down movements on the needle handle. Reducing method: (a) the needle body is not perpendicular to the plane of the meridian points (i.e., the included angle is less than 90°); (b) the middle finger nail is scraped up the needle handle with a strong force, but the downward force is small (i.e., weighing and scraping). Push gently. (c) After completing the stimulation of each meridian point, the thumb leaves the tail of the needle, while the middle finger keeps clamping the needle handle, and the needle tip is kept on the acupuncture point. Rotate six times in a counterclockwise arc. (3) Precautions: during the entire operation, the thumb should be kept lightly against the tail of the needle, so as not to increase the pressure involuntarily when the stimulation is applied and cause the needle to pierce the skin and cause pain. At the same time, when the middle finger nail is scraped and pushed on the needle handle, always keep the needle handle still in order to avoid irregular beating. The amplitude of the scraping and pushing should be large (that is, the full length of the needle handle is scraped and pushed), and the finger force must be soft and even; the speed should not be too fast or slow. The punctured part should always maintain a soft and even slight tremor, so that the patient can feel acupuncture and feel comfortable.

### 4.2. Clever Use of Acupoints to Embed Thread

The authors often use catgut embedding at acupoints to treat NAFLD, and the effect is stronger. Acupoint embedding therapy involves placement of absorbable surgical sutures into the viscera, meridians, and collaterals to infuse the Qi into a special part of the body surface, to produce continuous stimulation to the human body, so as to achieve the purpose of preventing and curing diseases. *Treatment Method*. (1) Acupoint selection: Zhongwan, Zusanli, Sanyinjiao, Fenglong, and Taichong. Point selection basis: Zhongwan is the mu point of the stomach meridian and the Fuhui of eight influential points. Zhongwan has the effect of regulating the spleen and stomach. Zusanli is the point of the stomach meridian. Zusanli can regulate the spleen and stomach and, when combined with Sanyinjiao, it can relieve the dampness of the Yangming Meridian and Taiyin Meridian and support the function of spleen and stomach transportation and transfusion. The Fenglong point is the luo point of the foot Yangming stomach meridian, and the point is also connected to the foot Taiyin spleen meridian. Therefore, this point can treat the two meridian disorders of the spleen and stomach and regulate Qi of the spleen and stomach meridian to ensure that the Qi is running and the body fluid is spreading. The spleen meridian governs transportation and transformation, and crosses out the wet and phlegm. The Taichong point is the original point of the liver meridian. It can soothe the liver, regulate the Qi, and regulate the channels of Qi. (2) Operation: the patient takes the supine position, the skin of the abdomen and lower limbs is fully exposed, and the acupoints are disinfected with Aner iodine or 75% alcohol. After washing and disinfecting the hands, the doctors put on sterile gloves. Needle selection: No. 7 injection needle (specification 0.7 mm × 32 mm) is used as a cannula, and an acupuncture needle (specification 0.30 mm × 50 mm) has the needle cut off as a needle core to make a simple embedding needle. Use sterile forceps to take the sterilized collagen thread with a length of 2 cm, place the needle tip into the injection needle, pierce the needle with the protein thread into the acupuncture point, push in the needle core, withdraw the needle tube, and pull the collagen thread. Embed the acupuncture points, put pressure on the needle hole with a cotton swab for a while after the needle is taken out, and then apply a sticking plaster to the needle hole. This is usually done once a week, for a total of 3 months of treatment. (3) Precautions: keep the needle eye position dry for 24 hours after embedding the thread to prevent infection.

### 4.3. Reusing Warm Acupuncture Treatment

**Figure 3:**
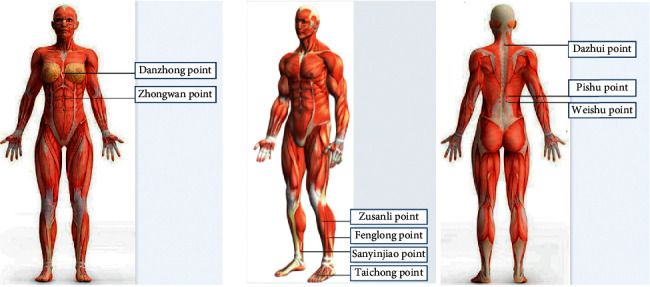
Acupoint location map.

Acupuncture and moxibustion treatment of NAFLD should focus on replenishing the spleen and stomach and treat the spleen from the perspective of “the spleen and stomach are the foundation of the acquired.” Therefore, warm acupuncture and moxibustion are chosen; this combines the dual effects of acupuncture and moxibustion, which has the effect of warming and invigorating the spleen Yang. Based on the theory of traditional Chinese medicine, Professor Wu pointed out that warming acupuncture or the moxibustion of strong points has the effect of replenishing spleen Yang, strengthening the body and eliminating pathology, so as to improve the immune function of the body [14]. Modern medicine shows that immune cells are also closely related to NAFLD. Both specific immunity and non-specific immunity are involved in the pathogenesis of NAFLD. The activation of immunogenic cells is positively correlated with the lipid content in the liver. The interaction between the endocrine system and immune cells may be an important mechanism for the occurrence and development of NAFLD [15]. *Treatment Method*. (1) Acupoint selection: Dazhui, Pishu, and Weishu. Point selection basis: Dazhui point is the meeting of the three Yang of the hands and feet and the governor channel, and it controls the Yang Qi of the whole body. Pishu is the beishu point of the spleen and Weishu is the beishu point of the stomach. They are the main points for the treatment of spleen and stomach diseases. Both belong to the tai Yang of the feet bladder meridian, so that they have a good effect of nourishing Yang. (2) Operation: the patient lies in the prone position, the skin on the back is exposed, and the acupuncture points are thoroughly disinfected. Routinely insert the needle, insert and twist, using the replenishing and reducing method; take a 2 cm long (one strong) moxa section, insert it on the needle, and light it. Moxibustion is performed for two strong moxibustions each time, and the needles are retained for 30 minutes. (3) Precautions: protect the skin with a piece of hard paper to prevent skin burns. If burned, apply moist scald cream (Figure 3).

## 5. Typical Case

A female patient XX, who was 35 years old, visited the doctor on May 11, 2020. Main complaint was fullness of the flanks for 2 weeks. Although the patient is a young woman, she feels obvious fatigue, and she sweats even more after exercise. She also reports anorexia, swelling, and excessive sleep. She is 157 cm tall, weighs 70 kg, and has a body mass index (BMI) of 28.4 kg/cm2. Previous physical examination revealed fatty liver for more than 1 year; the patient denied a history of viral hepatitis and other medical history and had no history of drinking alcohol. The tongue is pale, there are tooth marks on the sides, the fur is white and greasy, and the pulse is smooth. Western medicine diagnosis was non-alcoholic fatty liver disease; Chinese medicine diagnosis was liver pi (spleen deficiency, phlegm-dampness syndrome). Treatment method: invigorate the spleen and stomach and transport and resolve phlegm and dampness. Acupuncture methods used were shallow acupuncture, acupoint embedding, and warm needle moxibustion. Symptoms improved after 3 weeks, and after more than 3 months of treatment, the symptoms improved significantly, and the patient's weight decreased to 64.2 kg.

## 6. Summary

Acupuncture treatment of non-alcoholic fatty liver disease is simple and effective. The authors have conducted research on the treatment of NAFLD with acupuncture. The etiology and pathogenesis of NAFLD are based on “deficiency in origin and excess in superficiality”: deficiency in origin means deficiency of the spleen and stomach, and excess in superficiality is caused by hepatobiliary disorders.

Given that the spleen and stomach are the foundation of the acquired nature and that spleen deficiency and loss of transport are the internal basis of the disease, “treatment from the spleen, seeking the root of the disease” can show a miraculous effect. Acupuncture can regulate the Qi of the meridians and viscera to restore normal physiologic function. We summarize the characteristic therapies of traditional Chinese medicine acupuncture on the basis of our long-term clinical experience. We make good use of shallow acupuncture and acupoint embedding therapy to regulate the spleen and stomach, dredging the Qi of the whole body. Additionally, we reuse warm acupuncture for warming Yang, strengthening the spleen, and improving immune function. This study provides a reference for clinical treatment and provides a basis and foundation for the clinical promotion of acupuncture and moxibustion in the treatment of NAFLD.
